# Trivalent coronavirus vaccines elicit broad-spectrum immunity in mice and attenuate respiratory viral load in golden hamsters

**DOI:** 10.3389/fimmu.2026.1734382

**Published:** 2026-01-26

**Authors:** Xiuli Shen, Jiangshan He, Maoshun Liu, Xinyu Zhang, Peijia Wang, Yiming Shao

**Affiliations:** 1 State Key Laboratory for Diagnosis and Treatment of Infectious Diseases, The First Affiliated Hospital, School of Medicine, Zhejiang University, Hangzhou, China; 2 Changping Laboratory, Beijing, China; 3 School of Medicine, Nankai University, Tianjin, China

**Keywords:** coronaviruses, immune responses, immunogenicity, protective efficacy, trivalent vaccines

## Abstract

**Introduction:**

Coronaviruses frequently undergo genomic mutation and recombination in nature. Through cross-species infection and sporadic spillover events, novel coronaviruses may periodically emerge in humans. SARS-CoV-1, MERS-CoV, and SARS-CoV-2 all cause severe, predominantly respiratory diseases with moderate to high pathogenicity, posing a substantial threat to public health. To prepare for potential future coronavirus outbreaks, there is a need for universal vaccines capable of eliciting broad-spectrum humoral and cellular immunity.

**Methods:**

In this study, we constructed DNA- and replicating Vaccinia virus TianTan (VTT)-vectored monovalent and trivalent vaccines, using the spike (S) proteins of the aforementioned wild-type viruses as immunogens in a heterologous DNA-prime/VTT-boost regimen.

**Results:**

Compared with monovalent vaccines, the trivalent candidate induced robust, broad-spectrum humoral and cellular immune responses against the S proteins of SARS-CoV-1, MERS-CoV, and SARS-CoV-2 in mice. Notably, it also conferred protection against challenge with the SARS-CoV-2 XBB variant.

**Discussion:**

These findings offer important insights for developing practical multivalent coronavirus vaccines that could help mitigate transmission and mortality early in future coronavirus outbreaks. Such an initial countermeasure could buy critical time for the development of variant-specific vaccines and further inform the design of universal coronavirus vaccines.

## 1 Introduction

Coronaviruses are single-stranded positive-sense RNA viruses belonging to the genus Coronavirus within the family *Coronaviridae*. Phylogenetically, they are classified into four genera: *Alpha-, Beta-, Gamma-*, and *Deltacoronavirus* (1). Their genomes exhibit high variability and are prone to genetic recombination. Due to frequent cross-species transmission and sporadic viral spillover events, novel coronaviruses may emerge periodically in human populations. Epidemiological surveillance has identified nine human-pathogenic coronaviruses, spanning the *Alphacoronavirus* (HCoV-NL63, HCoV-229E and CCoV-HuPn2018) (2, 3), *Betacoronavirus* (HCoV-HKU1, HCoV-OC43, SARS-CoV-1, MERS-CoV and SARS-CoV-2) (4), and *Deltacoronavirus* (Hu-PDCoV) genera (5). Notably, *Betacoronavirus* has triggered three major respiratory disease outbreak events in the past two decades. SARS-CoV-1, which caused Severe Acute Respiratory Syndrome (SARS) in 2003, belongs to lineage B of the beta coronavirus genus. A total of 8,096 cases (with 774 deaths) were reported across 27 countries, resulting in a mortality rate of approximately 10% (6, 7). MERS-CoV, belonging to lineage C of the beta coronavirus genus, had a mortality rate of approximately 36% (8, 9). Cases of MERS-CoV infection have been reported in over 27 countries in the Middle East, Europe, North Africa, and Asia. SARS-CoV-2, which has spread widely worldwide, also belongs to lineage B of the beta coronavirus genus and shares 79.6% nucleotide similarity with SARS-CoV-1’s genome. Infected individuals may experience headaches, fever, runny nose, dry cough, dyspnea, myalgia, and fatigue, and may even develop gastrointestinal symptoms such as anorexia, vomiting, and diarrhea (10). Infected individuals exhibit increased levels of cytokines such as IL-6 and TNF-α, and severe cases may lead to a cytokine storm. Critically ill patients may develop acute respiratory distress syndrome, shock, metabolic acidosis, coagulation dysfunction, and other life-threatening conditions (11). The outbreaks caused by SARS-CoV-1, MERS-CoV, and SARS-CoV-2 pose severe challenges to human health (12, 13). Unfortunately, no formally approved vaccines are yet available for SARS-CoV-1 or MERS-CoV. Despite the significant achievements of COVID-19 vaccines, which have been emergency-approved by the WHO in controlling the pandemic, reducing severe cases, and lowering mortality rates, the prolonged battle against COVID-19 has given rise to multiple concerning Variants of Concern (VOCs). These VOCs can evade the protection offered by existing vaccines, leading to frequent breakthrough infections. The current COVID-19 vaccines have limited duration of immune protection and insufficient efficacy, making it difficult to provide broad-spectrum protection against newly emerging variant strains. Coronaviruses are single-stranded RNA viruses whose genomes are prone to mutation and recombination. Furthermore, the natural hosts of coronaviruses are widely distributed. Due to frequent cross-species infections and occasional spillover events, new strains of coronavirus may periodically emerge in humans (14). Therefore, the development of a universal vaccine capable of preventing or controlling infections from multiple coronavirus strains is regarded as a crucial strategy to eradicate this threat. Such a vaccine does not necessarily need to induce exceptionally potent humoral and cellular immune responses against every coronavirus variant. Instead, it must exhibit broad-spectrum efficacy capable of providing population-level immune protection during the early stages of a new outbreak. This would serve as a crucial interim measure, buying valuable time for the development of targeted, strain-specific vaccines.

After decades of development, vaccine technology has evolved into a diversified research and development system, mainly encompassing four major technical routes: inactivated vaccines (15), recombinant protein vaccines (16), nucleic acid vaccines (including mRNA vaccines) (17), and viral vector vaccines (18). Among these, inactivated vaccines retain the complete immunogenic structure of pathogens and can induce broad-spectrum immune responses against multiple antigenic epitopes, but they have limitations such as long production cycles and potential biosafety risks. Recombinant protein vaccines have become one of the mainstream technologies due to their high safety and low adverse reaction rate; however, they exhibit a single immunogenic epitope. Similar to inactivated vaccines, recombinant protein vaccines rely on immune adjuvants to enhance cellular immune responses, and the sustained-release effect of adjuvants prolongs the duration of antigen action in the body to improve immune efficacy. As a key branch of nucleic acid vaccines, mRNA vaccines are synthesized by *in vitro* transcription of antigen-encoding sequences, offering advantages such as short research and development cycles and flexible production processes. They can rapidly elicit humoral and cellular immune responses but are susceptible to RNA instability, imposing high requirements on delivery systems and storage conditions. Viral vector vaccines, by inserting target genes into the genome of vector viruses, enable efficient antigen expression in host cells through the infectivity of the vector, thereby inducing specific immune responses. Common vectors include vaccinia virus, adenovirus, influenza virus, and others. Such vector vaccines have been widely applied in the development of vaccines against human immunodeficiency virus (HIV), Middle East respiratory syndrome coronavirus (MERS-CoV), Zika virus, and severe acute respiratory syndrome coronavirus 2 (SARS-CoV-2), among others (19, 20). Notably, they demonstrate unique advantages in eliciting potent cellular immune responses (21), laying a solid foundation for the in-depth development of subsequent vector vaccines.

Vaccinia virus (VACV) is a large-sized, structurally complex double-stranded DNA virus, characterized by its unique replication process that occurs exclusively in the cytoplasm of mammalian cells. Endowed with key advantages including non-oncogenicity, excellent physicochemical stability, a broad host range, and a large genomic capacity, VACV serves as an ideal vaccine vector. Through homologous recombination technology, target foreign genes can be precisely inserted into the non-essential regions of the VACV genome, enabling efficient expression of the desired proteins in infected cells. Leveraging this property, VACV has been extensively utilized in the development of recombinant vaccines, such as Human Immunodeficiency Virus (HIV) vaccines and SARS-CoV-2 vaccines (18, 22). Notably, when combined with DNA vaccines, such vector vaccines can synergistically induce long-lasting and potent humoral immune responses, while eliciting robust cellular immune responses, thereby providing dual immune protection against intracellular pathogen infections. In this study, we developed a trivalent coronavirus DNA vaccine and a trivalent coronavirus VTT vaccine using spike proteins from SARS-CoV-1, MERS-CoV, and SARS-CoV-2 (Wuhan-Hu-1). We assessed the vaccine immunogenicity in BALB/c mice and evaluated its protective efficacy in golden hamsters.

## 2 Materials and methods

### 2.1 Coronavirus sequences

We queried amino acid sequences of spike protein from National Center for Biotechnology Information (NCBI). *Alphacoronavirus* (HCoV-229E, HCoV-NL63, BatAlpha-CoV and CCoV-HuPn2018), *Betacoronavirus* (HCoV-OC43, HCoV-HKU1, SARS-CoV-1, SARS-CoV-2, MERS-CoV and Bat-CoV-RaTG13), *Gammacoronavirus* (IBV, Canada goose-CoV and Duck-CoV), *Deltacoronavirus* (PD-CoV, PCoV-HKU15 and White eye-CoV HKU16).

### 2.2 Vaccines and animals

We focused on the spike (S) protein of various coronaviruses, including SARS-CoV-1, MERS-CoV, and SARS-CoV-2, as the antigenic target for vaccine development. The S proteins of coronaviruses were inserted into plasmid vectors to formulate individual DNA vaccines for each type of coronavirus. Analogously, the S protein of coronavirus was combined into the Vaccina virus TianTan (VTT) constructing VTT vaccines. Based on the differences in vector types and immunogen categories adopted by the vaccines, they are designated as DNA-SARS1, DNA-SARS2, DNA-MERS, VTT-SARS1, VTT-SARS2 and VTT-MERS respectively. The constructions of DNA and VTT vector vaccines were described in patents (CN101020055A and CN114032217A) and article (20). DNA vector vaccines containing spike proteins of SARS-CoV-1, SARS-CoV-2, and MERS-CoV coronaviruses were mixed at a 1:1:1 ratio to prepare a trivalent DNA vector vaccine; similarly, VTT vector vaccines containing spike proteins of SARS-CoV-1, SARS-CoV-2, and MERS-CoV coronaviruses were mixed at a 1:1:1 ratio to prepare a trivalent VTT vector vaccine.

The immunogenicity assessment of the vaccines was conducted on 6 to 8 weeks old female BALB/c mice, which were maintained under specific pathogen-free (SPF) conditions. Furthermore, the efficacy of these vaccines in conferring immune protection was validated through the utilization of 6-week-old Lvov’s Golden Syrian hamsters (LVG golden hamsters). BALB/c mice and *LVG golden hamsters* were purchased from Beijing Vital River Laboratory (Beijing, China).

### 2.3 Animals immunization and XBB challenge experiments

To verify the immunogenicity and immune protection effect of the vaccines, we have designed two immunization protocols.

Firstly, BALB/c mice in the vaccine groups (n=5) were intramuscularly (i.m.) immunized with coronavirus DNA vaccines via the electroporation method (electroporation parameters: 100 V, 60 ms, interval 999 ms, 10 times; WJ-2005-Intelligent live cell gene delivery instrument, NINGBO SCIENTZ BIOTECHNOLOGY CO.LTD) at days 0 and 14, with a dose of 50 μg per mouse per immunization, administered to the tibialis anterior muscles; Subsequently, the mice were intramuscularly (i.m.) boosted with recombinant VTT vaccines at day 70, with a dose of 5×10^6^ plaque-forming units (pfu) per mouse. However, the mice in control group (n=3) were immunized empty DNA vector and VTT. On the 14th day after the end of immunization, the blood and spleens of the BALB/c mice were collected to detect humoral and cellular immune responses respectively.

The second experimental protocol was designed to validate the protective effect of the trivalent vaccine in LVG golden hamsters, with twelve animals per group (6 females and 6 males). The immune doses of DNA and recombinant VTT vaccines are 50 μg and 1×10^7^ pfu per mouse each time respectively. 14 days after immunization, the golden hamsters were challenged with the XXB strain (SARS-CoV-2 XBB RY166, 6.125lgCCID50/ml) via the intranasal. Following viral challenge, the body weight of hamsters in the vaccine groups and the PBS control group (n=6 per group; 3 females and 3 males) was recorded daily, and nasopharyngeal swabs were collected from these hamsters for viral load detection. On the other hand, after the XBB challenge, on days 3, 5, and 7, two LVG golden hamsters per group (1 female and 1 male) were sacrificed, and their bronchial tissues and lung tissues were collected for viral load detection.

### 2.4 Enzyme-linked immunosorbent assay

The specific binding antibodies were detected using enzyme-linked immunosorbent assay (ELISA). The high affinity plates (Corning) were coated with coronavirus receptor binding domain (RBD) protein 100 μL per well (1.5μg/mL) overnight at 4°C and then blocked with 5% skim milk-2% bovine serum albumin (23). The immunized sera were diluted in series and then added into the coated plates with 100 μL per well. HRP-conjugated goat anti-mouse ((Southern Biotech) was diluted at 1:2500 to be used in ELISA experiments. Color development was achieved using TMB as the substrate (Beijing Kinghawk Pharmaceutical), and the reaction was terminated after 10 minutes. Optical density was detected using dual wavelengths of 450nm and 460nm. Endpoint titers with an absorbance over two times that of the control group were considered positive.

### 2.5 Pseudovirus neutralization assays

The neutralizing antibody levels were determined by pseudovirus neutralization assay. The construction of vesicular stomatitis virus (VSV)-based pseudoviruses for coronaviruses was based on previous literature (24). The inactivated immune sera were serially diluted and then incubated with pseudoviruses at 37°C for 1 hour. Subsequently, 100 μL of Huh7 cells (2×10^5^ cells/well) were added to each well and cultured under 37°C, 5% CO_2_ culture conditions for 24 hours. The neutralizing titers at 50% (NT50) were detected using Ultra-High Sensitivity luciferase reagents (PerkinElmer, MA) by a chemiluminescence detector (VICTOR3, PerkinElmer). The values of NT 50 were calculated by the Reed-Muench method.

### 2.6 Enzyme-linked immunospot assay

The specific T cell responses were accessed using the mouse IFN-γ ELISpot assay set (BD Biosciences). The ELISpot plates were coated with purified anti-mouse IFN-γ (5μg/mL) overnight at 4°C and then blocked for 2 hours at room temperature (RT). Fresh mouse splenocytes were seeded in the blocked ELISpot plates, and the splenocytes from each group of mice were stimulated with three coronavirus peptide pools, respectively (20, 22). The positive control was stimulated with cell stimulation cocktail (Invitrogen), and the negative control group was stimulated with dimethyl sulfoxide (DMSO) (Sigma). The splenocytes were cultured with the peptide pools for 18 hours, washed, and then incubated with biotinylated anti-mouse IFN-γ (2μg/mL) for 2 hours at RT according to the instruction manual. Subsequently, the streptavidin-HRP (1:100) were added into the plates and incubated an hour. Finally, the 3-Amino-9-ethylcarbazole (AEC) substrate set (BD Biosciences) was used for chromogen in ELISpot assay. The results were counted using the ELISpot reader instrument (AID GmbH).

### 2.7 Analysis of viral load by quantitative RT-PCR

To assess protective efficacy of the vaccines on golden hamsters, we measured the levels of viral load in pharyngeal swabs, lung tissues (part of the right lung), and tracheas of golden hamsters after virus challenge. The tissue samples underwent homogenization, followed by centrifugation to separate the supernatant. Subsequently, the total RNA was extracted from the tissue supernatant. The total RNA was extracted from tissues using the Fastpure Cell/Tissue Total RNA Isolation Kit V2 (Vazyme). The TaqPath™ 1-Step RT-qPCR Master Mix, CG (Thermo Fisher Scientific Inc.) kit was employed to detect the levels of viral load. The system and procedure for PCR reactions followed the instruction manual. Viral load was detected using the LightCycler 480 II Real-Time Fluorescent Quantitative PCR instrument (Roche). Primers and probes were synthesized by Beijing Tsingke Biotechnology Co., Ltd. (prime-F: TTACAAACATTGGCCGCAAA, prime-R: GCGCGACATTCCGAAGAA and probe: FAM-ACAATTTGCCCCCAGCGCTTCAG-BHQ1).

### 2.8 Statistical analyses

The antibody levels, neutralizing antibody titers, and viral load levels were described using geometric means, whereas the results of cellular immune responses were presented using mean values. The statistical analyses were performed using the Kruskal-Wallis test followed by the Dunn’s multiple comparisons test. The GraphPad Prism 9.5.1 software was utilized for plotting, and statistical significance was indicated by p values (*p < 0.05, **p < 0.01, or ***p < 0.001).

## 3 Results

### 3.1 The trivalent coronavirus vaccine generates broad-spectrum and robust humoral immunity

**Figure 1:**
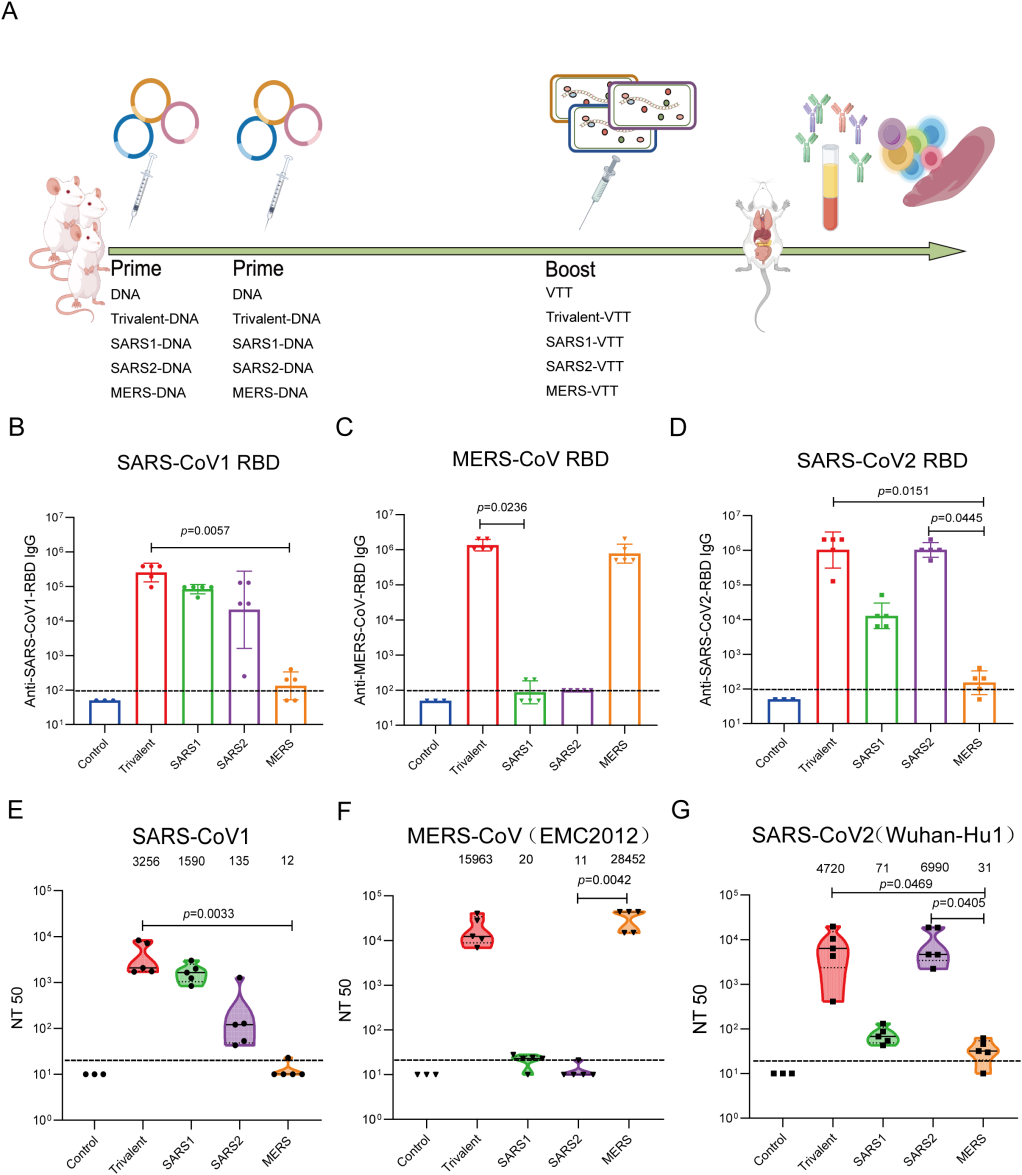
Mice immunized with monovalent vaccines and trivalent vaccine developed humoral immune responses. **(A)** Experimental protocol for vaccine immunogenicity. Binding antibody titers in immune sera against SARS-CoV-1 RBD **(B)**, SARS-CoV-2 RBD **(C)**, and MERS-CoV RBD **(D)** were detected using ELISA. Neutralizing antibody titers in immune sera against pseudovirus of SARS-CoV-1 **(E)**, SARS-CoV-2 **(F)**, and MERS-CoV **(G)** were determined using a neutralization assay. Data were shown as geometric means ± SD and analyzed using the Kruskal-Wallis test and the Dunn’s multiple comparisons test.

To assess the immunogenicity of the vaccine, BALB/c mice were primed with DNA vaccines and then boosted with VTT vaccines (DNA-prime/VTT-boost strategy). Subsequently, the humoral and cellular immune responses in the mice were evaluated 14 days post the third vaccination. The DNA vaccination was administered with electroporation (electroporation parameters: 100V, 60ms, with an interval of 999ms, repeated 10 times) (**Figure 1A**).

Humoral immune responses were assessed by measuring levels of binding antibodies and neutralizing antibodies. We detected the IgG antibody titers of immunized sera against SARS-CoV-1-RBD, MERS-CoV-RBD, and SARS-CoV-2-RBD via ELISA. The trivalent-DNA/VTT group, SARS1-DNA/VTT group, and SARS2-DNA/VTT group demonstrated specific binding antibodies against SARS-CoV-1-RBD, with geometric mean titers (GMTs) of 271,529, 76,195, and 20,159, respectively. In contrast, the SARS-CoV-1-RBD-specific binding antibody titer in the MERS-DNA/VTT group was only 126. The GMT of SARS-CoV-1-RBD-specific binding antibodies was highest in the trivalent vaccine group, significantly higher than that in the MERS-DNA/VTT group (P = 0.0057) (**Figure 1B**). Trivalent-DNA/VTT and MERS-DNA/VTT groups exhibited robust specific binding antibody responses against MERS-CoV-RBD protein, with geometric mean titers (GMTs) of 912,280 and 724,077, respectively. In contrast, the SARS1-DNA/VTT and SARS2-DNA/VTT groups elicited significantly lower specific binding antibody responses against MERS-CoV-RBD protein, with GMTs of 178 and 112, respectively. Statistical analysis revealed that the GMT of specific binding antibodies against MERS-CoV-RBD protein in the Trivalent-DNA/VTT group was significantly higher than that in the SARS1-DNA/VTT group (P = 0.0236) (**Figure 1C**). The results indicated that both the Trivalent-DNA/VTT and SARS2-DNA/VTT groups exhibited high titers of SARS-CoV-2-RBD-specific binding antibodies, with geometric mean titers (GMTs) reaching 1,024,000 and 1,149,401 respectively. In contrast, the SARS1-DNA/VTT group displayed moderate levels of SARS-CoV-2-RBD-specific binding antibody, with a GMT of 11,404. Notably, the MERS-DNA/VTT group demonstrated the lowest level of SARS-CoV-2-RBD-specific binding antibodies, exhibiting a GMT of merely 126, which was significantly lower than that observed in the trivalent-DNA/VTT and SARS2-DNA/VTT groups (P = 0.0151 and P = 0.0445, respectively) (**Figure 1D**). The results showed that the trivalent vaccine group developed strong and broad-spectrum binding antibody activities, while the monovalent vaccine groups only elicited a high level of binding antibody response against themselves.

To further investigate the humoral immune responses induced by the vaccines, we also assessed the levels of neutralizing antibodies in the mice. The trends of neutralizing antibodies in the vaccine groups were similar to those of binding antibodies (**Figures 1E–G**). The trivalent-DNA/VTT group exhibited robust neutralizing responses against SARS-CoV-1, SARS-CoV-2, and MERS-CoV, with neutralizing antibody GMTs of 3256, 4720, and 15963, respectively. In contrast, the SARS1-DNA/VTT group elicited low titers of neutralizing antibodies against SARS-CoV-2 and MERS-CoV, with GMTs of only 20 and 71, respectively. The SARS2-DNA/VTT group, while effective in generating neutralizing antibody against SARS-CoV-2, failed to neutralize MERS-CoV and displayed limited neutralizing capacity against SARS-CoV-1(GMT = 135). Conversely, the MERS-DNA/VTT induced robust neutralizing responses against MERS-CoV (GMT = 28,452) but exhibited virtually no neutralizing activity against SARS-CoV-2 and SARS-CoV-1. Statistical analysis revealed that the GMTs of neutralizing antibodies against SARS-CoV-1 and SARS-CoV-2 in the Trivalent-DNA/VTT group were significantly higher than those in the MERS-DNA/VTT group (P = 0.0033, P = 0.0469, respectively) (**Figures 1E-G**). Furthermore, the neutralizing responses against MERS-CoV in MERS-DNA/VTT group were markedly higher compared to those from the SARS2-DNA/VTT group (P = 0.0042). Notably, the neutralizing reactions against SARS-CoV-2 in the SARS2-DNA/VTT group were significantly higher than those in the MERS-DNA/VTT group (P = 0.0405). The trivalent vaccine demonstrated robust neutralizing capability against three types pseudoviruses of SARS-CoV-1, SARS-CoV-2, and MERS-CoV, whereas the neutralizing antibodies elicited by monovalent vaccines exhibited comparable neutralizing potency to homologous pseudoviruses as those by the trivalent vaccine, yet significantly weaker neutralizing capacity against heterologous pseudoviruses.

### 3.2 The trivalent coronavirus vaccine generates strong cellular immunity

**Figure 2:**
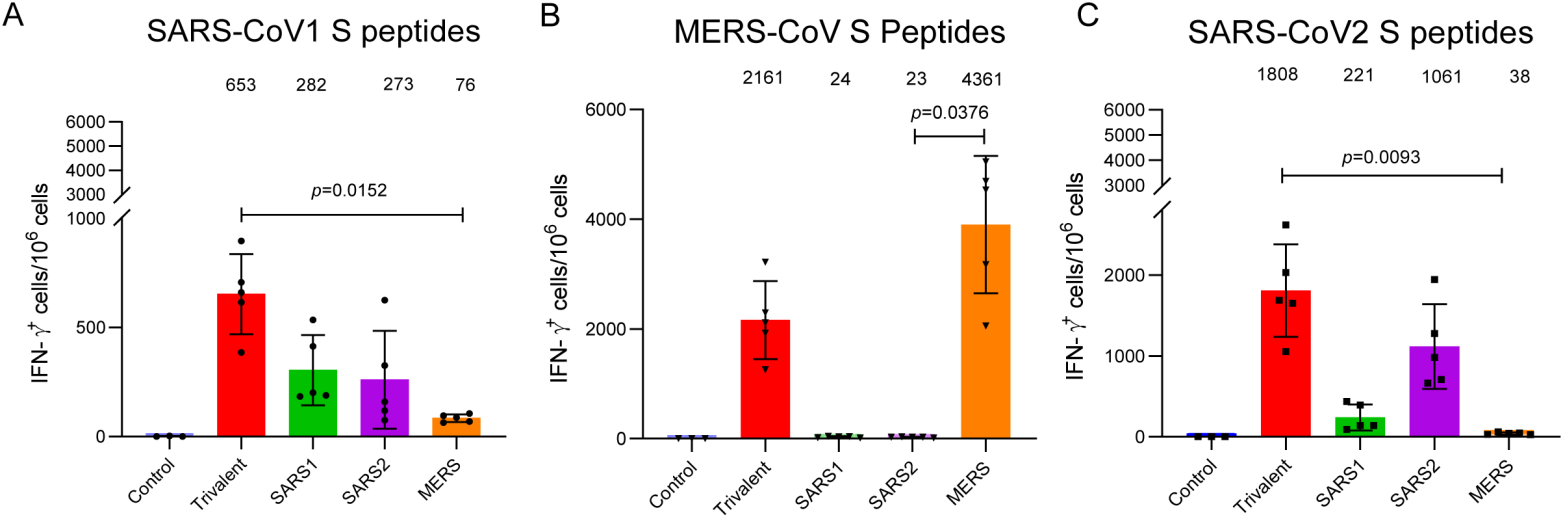
Mice immunized with monovalent vaccines and the trivalent vaccine generated specific cellular immune responses. The intensity of cellular immune responses in mouse splenocytes from mice against SARS-CoV-1 **(A)**, SARS-CoV-2 **(B)**, and MERS-CoV **(C)** was detected using the ELISPOT assay. Data were shown as arithmetic means ± SD and analyzed using the Kruskal-Wallis test and the Dunn’s multiple comparisons test.

The specific T cell responses were measured using an ELISpot assay. The trivalent-DNA/VTT group exhibited moderate levels of IFN-γ^+^ T cells (653 spot forming unit/10^6^ splenic lymphocytes) against the SARS-CoV-1 S peptide pool, which were 2.32-fold (282 SFU/10^6^ splenic lymphocytes) and 2.39-fold (273 SFU/10^6^ splenic lymphocytes) higher than those induced by the SARS1-DNA/VTT and SARS2-DNA/VTT groups, respectively. Notably, the specific T cell response against the SARS-CoV-1 S peptide pool in the trivalent-DNA/VTT group was significantly stronger than that of MERS-DNA/VTT group (P = 0.0152), which had only 76 SFU/10^6^ splenic lymphocytes (**Figure 2A**). Both the trivalent and monovalent MERS vaccines immunized mice elicited robust MERS-CoV S-specific IFN-γ^+^ T cell responses in immunized mice. In contrast, SARS1-DNA/VTT and SARS2-DNA/VTT groups barely induced MERS-CoV S -specific IFN-γ^+^ T cell responses in mice. Specifically, the MERS-CoV S-specific IFN-γ^+^ T cell response induced by the MERS-DNA/VTT group was statistically different from that of the SARS2-DNA/VTT groups (P = 0.0376, respectively) (**Figure 2B**). The trivalent-DNA/VTT group generated strongly specific IFN-γ^+^ T cell responses targeting the S protein of SARS-CoV-2, which were significantly higher than that observed in the MERS-DNA/VTT group (P = 0.0093). While the SARS1-DNA/VTT group induced a lower level of specific IFN-γ^+^ T cell response against the SARS-CoV-2 S peptide pool, amounting to 221 SFU/10^6^ splenic lymphocytes. Notably, mice immunized with the MERS-CoV vaccines elicited barely any specific IFN-γ^+^ T cell response against the SARS-CoV-2 S peptide pool (**Figure 2C**). Compared to monovalent vaccines, the trivalent vaccine elicited strong specific IFN-γ^+^ T cell responses targeting the S proteins of SARS-CoV-1, MERS-CoV, and SARS-CoV-2.

### 3.3 The trivalent coronavirus vaccine induces broad-spectrum humoral and cellular immune responses

**Figure 3:**
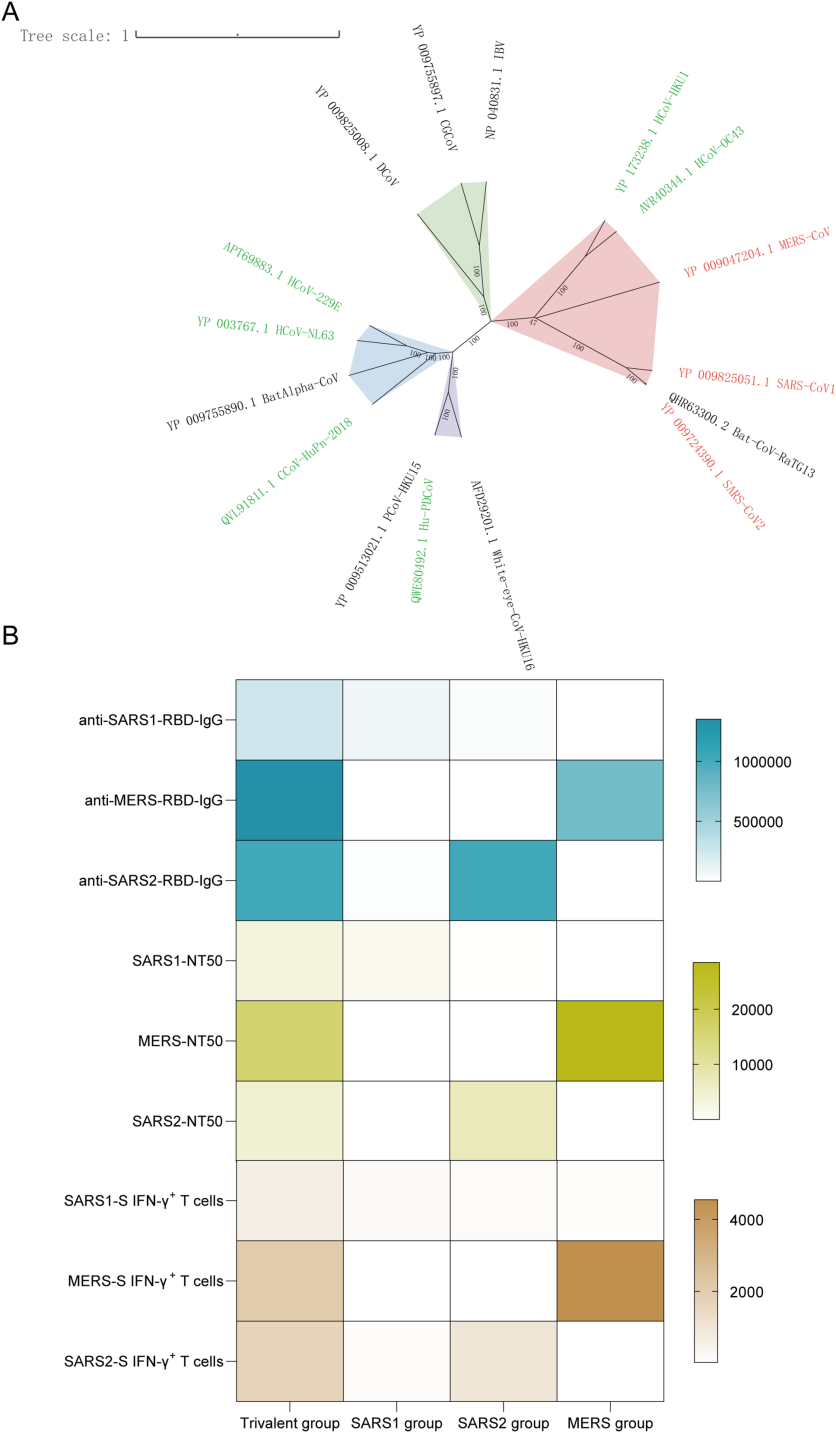
**(A)** Phylogenetic tree of SARS-CoV-1, SARS-CoV-2, and MERS-CoV based on full-length spike (S) protein amino acid sequences. **(B)** The heatmap showed the profiles of humoral and cellular immune responses in the vaccinated group.

To analyze suitable coronavirus S proteins as immunogens, we retrieved S protein amino acid sequences from representative viruses of the four coronavirus genera (*Alpha, Beta, Gamma, Delta*) in the NCBI database and conducted phylogenetic analysis. Nine coronaviruses known to cause human respiratory diseases belong to the *Alpha* (light green in the phylogenetic tree), *Beta* (light red) and *Delta* (purple) genera. Notably, the viruses - SARS-CoV-1, MERS-CoV, and SARS-CoV-2 are the *Beta genus* and exhibit close genetic relatedness (**Figure 3A**). Accordingly, we have constructed a trivalent vaccine targeting these three viruses for preparedness against potential future human coronavirus outbreaks. Trivalent coronavirus vaccines immunize mice using a Prime/Boost strategy, which not only elicits immune responses against a single viral strain but also covers different subtypes within the same genus (**Figure 3B**). This provides crucial evidence for the development of broad-spectrum vaccines and holds significant application value, particularly in addressing viral variations and emerging coronavirus threats.

### 3.4 The trivalent coronavirus vaccine attenuates respiratory viral load in Golden Hamsters following SARS-CoV-2 XBB (RY166) challenge

**Figure 4:**
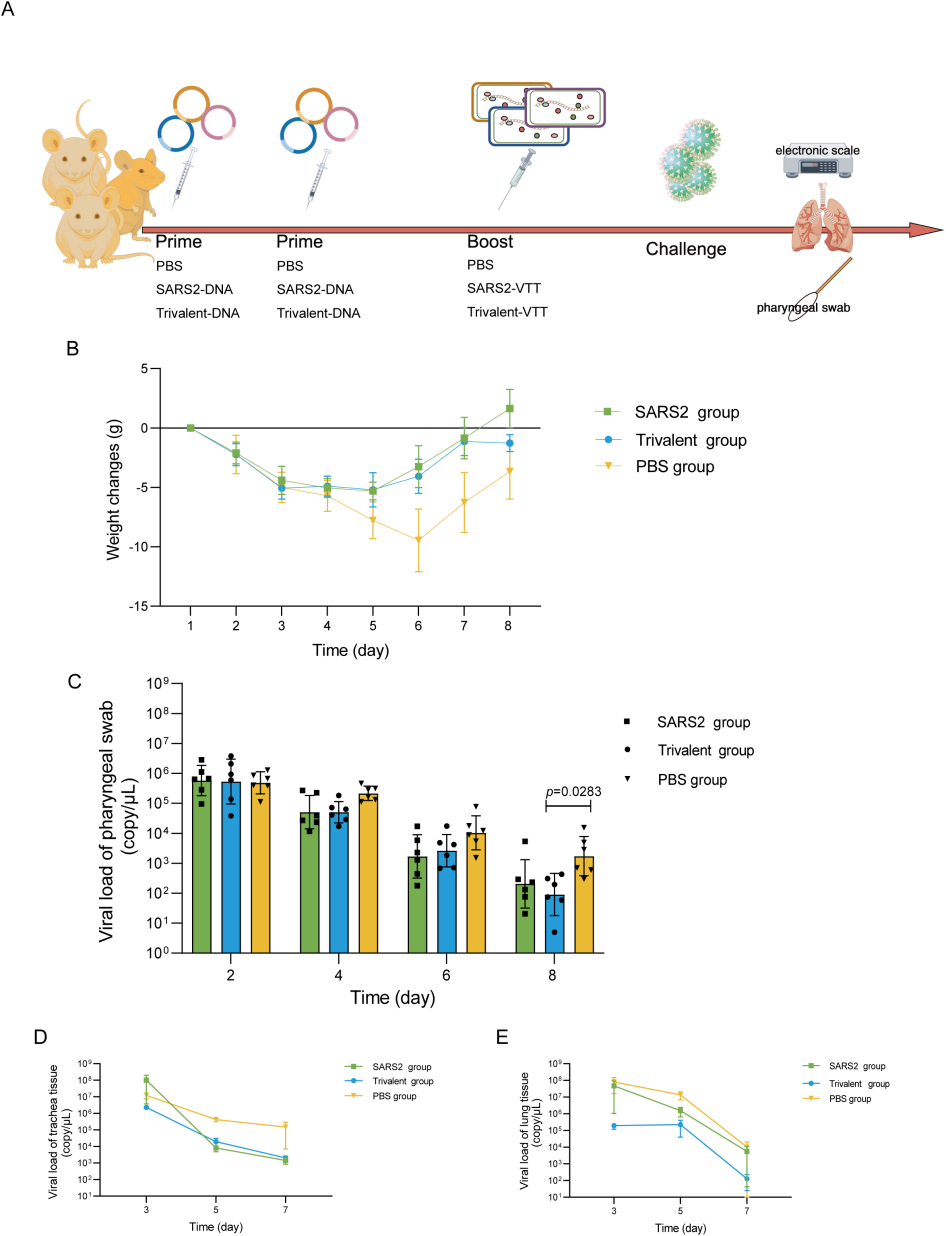
The coronavirus vaccines alleviated weight loss and reduced respiratory viral loads in the vaccinated group. **(A)** The scheme of challenge experiment. **(B)** Weight loss of mice in various groups was shown after challenge. Viral loads in pharyngeal swabs **(C)**, tracheal tissue **(D)**, and lung tissue **(E)** were detected using RT-qPCR after the challenge. Data were shown as geometric means ± SD and analyzed using the Kruskal-Wallis test and the Dunn’s multiple comparisons test.

The golden hamsters were vaccinated with DNA vaccines at week 0 and week 2, and then boosted with VVT vector vaccines at week 6. Following challenge with the SARS-CoV-2 XBB (RY166) at week 8, **Figure 4A**. Starting from the second day after challenged with the SARS-CoV-2 XBB (RY166), the body weight of the hamsters began to decline. In the vaccine groups, the hamsters’ body weight ceased to decline on the fifth day and started to increase on the sixth day. In contrast, the hamsters in the PBS control group experienced a persistent decline in body weight, initiating from the second day and continuing until the sixth day, with a weight reduction nearly 10% (**Figure 4B**). The results indicated that, starting from the fourth day post-challenge, the viral load in the pharyngeal swabs of hamsters began to decline. Compared to the PBS control group, a more reduction in viral load was observed in the pharyngeal swabs of hamsters in the vaccine group (**Figure 4C**). On the eighth day, the geometric mean titer (GMT) of viral load in the trivalent vaccine group dropped to 91 copies/mL. The viral load GMT in the PBS group was approximately 19-fold higher than that in the trivalent vaccine group (P = 0.0283) and was more than 8-fold higher than that in the COVID-19 vaccine group. After challenge with SARS-CoV-2 XBB (RY166), the vaccine group of hamsters exhibited a fast decline in virus load in the trachea and lung tissues compared to the control group (**Figures 4D, E**).

### 3.5 The trivalent coronavirus vaccine attenuates pulmonary pathological lesions in SARS-CoV-2 XBB (RY166)-challenged golden hamsters

**Figure 5:**
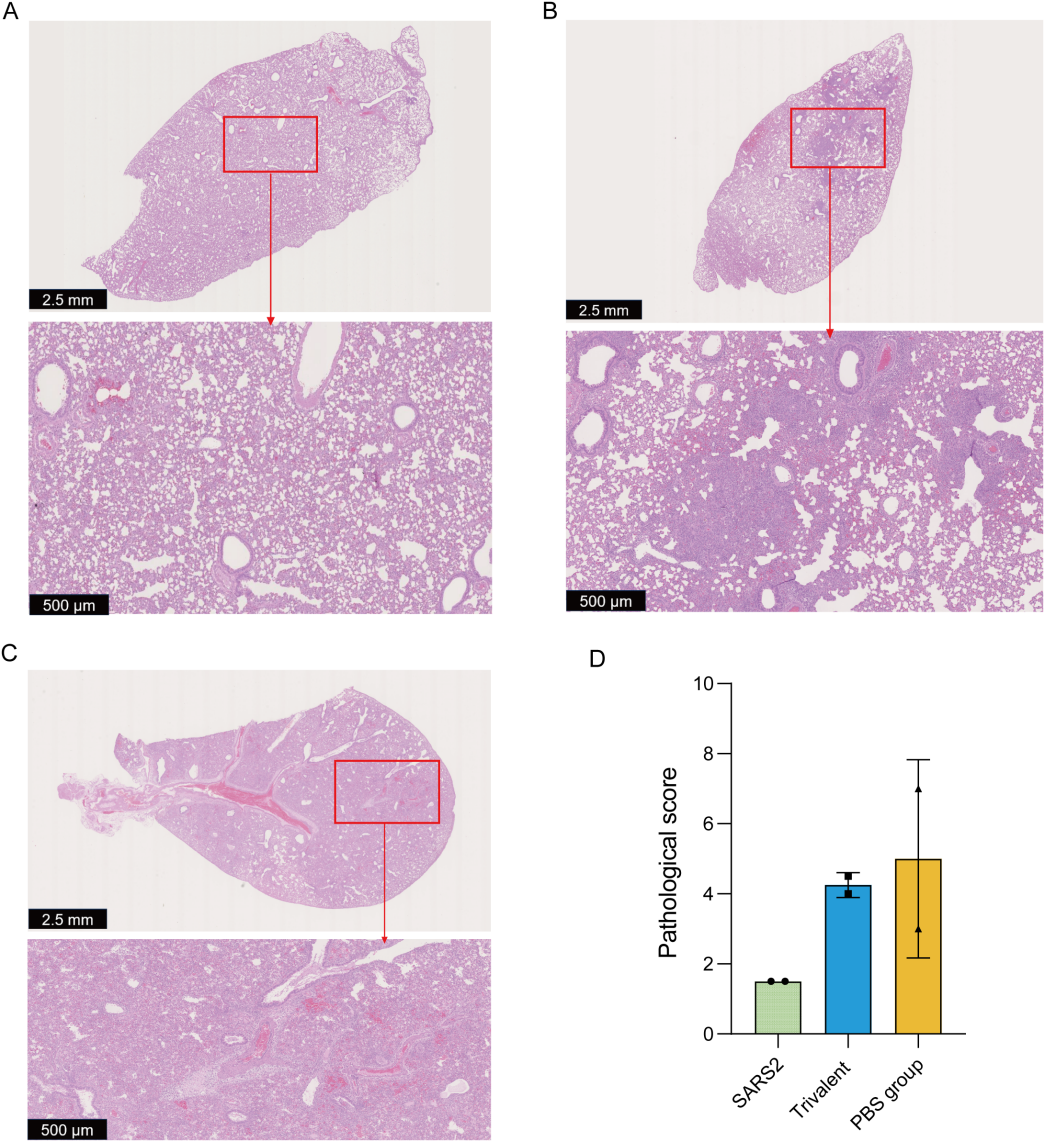
The coronavirus vaccines attenuate pulmonary pathological lesions in the vaccinated group. **(A)** Pathological sections of Golden Hamsters in the SARS2-DNA/VTT group. **(B)** Pathological sections of Golden Hamsters in the trivalent-DNA/VTT group. **(C)** Pathological sections of Golden Hamsters in the PBS group. **(D)** Pathological scores of the three experimental groups.

After challenge with the SARS-CoV-2 XBB variant, golden hamsters exhibited the maximum body weight loss on days 5–6 post-infection, therefore, lung tissue samples on day 5 were used for pathological section examination. Overall, among the three experimental groups, the PBS control group exhibited severe pulmonary pathological lesions, characterized by marked thickening of the alveolar septa and a substantial infiltration of monocytes and lymphocytes (**Figure 5A**). In contrast, the SARS2-DNA/VTT group exhibited the mildest pathological damage: the epithelial structures of the bronchi and bronchioles remained largely intact, with only mild inflammatory manifestations such as focal infiltration of monocytes and lymphocytes observed in the alveolar septa (**Figure 5B**). As for the Trivalent-DNA/VTT group, multiple small patchy pulmonary consolidations were detected, and the extent of inflammatory injury was slightly more severe than that in the SARS2-DNA/VTT group (**Figure 5C**). Collectively, these results demonstrate that vaccination can effectively mitigate lung injury induced by the SARS-CoV-2 XBB variant in golden hamsters (**Figure 5D**).

## 4 Discussion

SARS-CoV-1, MERS-CoV, and SARS-CoV-2 within the genus Beta-coronavirus are closely related in terms of phylogenetic relationship, all of which can cause severe respiratory diseases in humans. In this study, the S proteins of these three coronaviruses were selected as immunogens to construct trivalent coronavirus DNA vaccines and VTT vaccines. The DNA-Prime/VTT-Boost immunization strategy was adopted to immunize Babl/c mice, and the titers of specific binding antibodies and neutralizing antibodies against the three viruses in their immune sera were detected. The results showed that after immunizing mice with the trivalent coronavirus vaccine, the induced binding antibody and neutralizing antibody responses against SARS-CoV-1-RBD, MERS-CoV-RBD, and SARS-CoV-2-RBD were all strong; in contrast, the spectrum of binding antibodies and neutralizing antibodies induced by immunizing with SARS-CoV-1, MERS-CoV, or SARS-CoV-2 vaccine alone was relatively narrow. A mosaic nanoparticle vaccine displaying eight coronavirus RBDs (mosaic-8) was immunized in mice without diminishing the immune response against any specific RBD (25). Compared to homotypic SARS-CoV-2 nanoparticles vaccine, this heterotypic mosaic vaccine induced higher antibody titers against mismatched RBDs, this represents another finding supporting the co-display approach for inducing broader anti-coronavirus responses. Moreover, the heterotypic mosaic vaccine co-display strategy demonstrated advantages for eliciting neutralizing antibodies against zoonotic sarbecoviruses, thus potentially also providing protection against emerging coronaviruses with human spillover potential (26). Highly variable viruses (e.g., SARS-CoV-2, influenza viruses, HIV) continuously evolve through genetic mutations, recombination, and natural selection, posing multiple challenges for preventive vaccine development. Compared to single immunogen approaches, multivalent vaccines demonstrate significant advantages. Multivalent vaccines developed through protein-based, mRNA, and other technological platforms can elicit broader and more potent immune responses (27–29). The decavalent vaccine incorporating seasonal influenza viruses, avian influenza viruses, and SARS-CoV-2 variants elicited robust immune responses in immunized mice, generating binding antibodies, neutralizing antibodies, and antigen-specific cellular immune responses against all vaccine-matched influenza viruses and SARS-CoV-2 strains. In murine models, the vaccine provided complete protection against both homologous and heterologous strains of influenza viruses and SARS-CoV-2. The results highlight the potential of multivalent vaccines.

Coronaviruses have coexisted with humans for extended periods. Emerging evidence indicates that SARS-CoV-2-specific T cell responses play a critical role in viral clearance, infection prevention, the establishment of durable immune memory, and the recognition of viral variants. Vaccination similarly induces such responses, which likely represent the core mechanism underlying protection against severe disease and death (30, 31). Our study demonstrates that the DNA-prime/VTT-boost immunization strategy elicits a balanced immune response in mice. The research findings on other VTT vector vaccines are consistent with ours, all indicating that such vaccines can induce a balanced immune response (32). Compared to monovalent vaccines, the trivalent vaccine not only induces broad-spectrum humoral immunity against SARS-CoV-1, MERS-CoV, and SARS-CoV-2 but also triggers virus-specific cellular immune responses targeting these coronaviruses. T cell immune responses mediate vaccine protection (33), K18-hACE2 mice immunized with the Sarbeco RBD Mix vaccine and depleted of CD8^+^ T cells showed comparable lung viral RNA levels to controls upon SARS-CoV-2 B.1.351 challenge, indicating that CD8^+^ T cells are essential for vaccine-mediated protection in the lung. T cell depletion delayed viral clearance in the nasal turbinates and, to a lesser degree, in the lungs, underscoring their role in infection control, the studies aligned with the contribution of prior infection in generating protective T cell responses (34, 35).

Coronavirus vaccination confers cross-protection against seasonal human coronaviruses (HCoVs) in most individuals, a phenomenon attributed to the antigenic cross-reactivity of the S protein between SARS-CoV-2 and seasonal HCoVs (36, 37). Specifically, the highly conserved regions within the S antigen shared among HCoVs serve as key targets for cross-reactive antibodies; notably, such cross-reactive anti-S IgG antibodies recognize epitopes not only in the S2 subunit but also in the S1 subunit of the S protein. Against this mechanistic backdrop, we constructed a trivalent coronavirus vaccine and systematically compared its immunogenicity with that of monovalent vaccine counterparts. Distinguished by a broader repertoire of immunogens. Upon subsequent encounter with homologous or heterologous antigens carrying identical or conserved epitopes, the pre-established memory pool rapidly activates the primed immune system to mount a potent recall response. Collectively, these characteristics endow the trivalent vaccine with a distinct immunological mechanism that underpins its potential to elicit cross-reactive immunity against multiple coronavirus variants.

In our study, a challenge experiment was conducted by intranasal infection with the XBB strain on golden hamsters immunized with the trivalent vaccine and single coronavirus vaccine via the DNA-Prime/VTT-Boost strategy. The results showed that the degree of weight loss in golden hamsters in both the trivalent vaccine group and the single coronavirus vaccine group was lower than that in the PBS control group, and the viral load in tissues such as throat swabs and trachea was also lower than that in the control group. These results indicated that both the trivalent vaccine and the single coronavirus vaccine can enhance the resistance of golden hamsters to XBB strain attack. The trivalent coronavirus vaccine can alleviate severe pulmonary pathological damage induced by SARS-CoV-2 XBB variant infection, which is fully consistent with the original design intent of the vaccine. Its core objective is not to block viral infection, but to prevent severe pneumonia caused by infection. Therefore, this vaccine can be deployed as an emergency prevention option when specific vaccines targeting this variant have not yet been developed successfully. Over the past five years, coronaviruses have undergone continuous evolution, leading to the emergence of multiple variants capable of evading immune protection conferred by existing vaccines (38) or neutralizing antibodies (39–41). Key variants demonstrated significant mutations in the spike protein, particularly in the receptor-binding domain (RBD), resulting in reduced neutralization by vaccine-induced antibodies. This immune escape limited the effectiveness of COVID-19 vaccines. Multivalent vaccines against SARS-CoV-2 (42–44), multivalent vaccines targeting respiratory viruses (45, 46), and multiple multivalent candidate vaccines against coronaviruses (47, 48) are currently at different stages of development and have achieved phased progress. Especially, Bivalent COVID-19 booster vaccines have demonstrated robust immunogenicity and favorable safety profiles in both preclinical studies and early-stage clinical trials, emerging as a critical countermeasure against the persistent antigenic evolution of coronaviruses (49, 50). Our research findings provide important insights for future defense measures against coronavirus outbreaks and offer referenceable scientific data and immunization strategies for the development of universal coronavirus vaccines.

This study has certain limitations: due to resource constraints, the immune protective efficacy of the trivalent vaccine against SARS-CoV-1 and MERS-CoV has not yet been evaluated. Nevertheless, in conclusion, compared with monovalent vaccines, the trivalent coronavirus vaccine, when administered to mice using the DNA-Prime/VTT-Boost immunization strategy, can induce robust and broad-spectrum humoral and cellular immune responses, and confer protective effects against challenge with the SARS-CoV-2 variant strain XBB.

In conclusion, this study demonstrates that a trivalent vaccine based on wild-type immunogens can induce robust, broad-spectrum humoral and cellular immune responses against SARS-CoV-1, MERS-CoV, and SARS-CoV-2, and provides protection against challenge with the currently circulating SARS-CoV-2 XBB variant. Our findings support a two-step vaccination strategy: using multivalent coronavirus vaccines early in an outbreak to reduce transmission and mortality, thereby buying critical time for the development of variant-specific vaccines. This approach also offers valuable insights for the future design of universal coronavirus vaccines.

## Data Availability

The original contributions presented in the study are included in the article/supplementary material. Further inquiries can be directed to the corresponding author.
